# Immune checkpoint inhibitor-induced cardiotoxicity: from immune mechanisms to clinical surveillance and targeted therapies

**DOI:** 10.3389/fcvm.2026.1872588

**Published:** 2026-07-29

**Authors:** Nathan K. Chen, Kathryn D. Hok, Narsi Chokshi, Anthony Shadid, Haydn E. Rich, Lavanya Gunamalai, Marie-Francoise Doursout, Nirmal K. Banda, Pooja Shivshankar

**Affiliations:** 1 Institute of Molecular Medicine, UTHealth-McGovern Medical School, Houston TX, United States; 2 Department of Anesthesiology, UTHealth-McGovern Medical School, Houston TX, United States; 3 Division of Rheumatology-Department of Medicine, School of Medicine, University of Colorado Anschutz Medical Campus, Aurora, CO, United States

**Keywords:** arrhythmias, cardio-oncology, cardiotoxicity, complement immunity, immune checkpoint inhibitors, immunotherapy, myocarditis, PD-1/PD-L1

## Abstract

Immune checkpoint inhibitors (ICIs) have transformed the treatment landscape across a broad range of malignancies by restoring antitumor immunity through blockade of key inhibitory immune pathways, including programmed death receptor-1 (PD-1), programmed death ligand-1 (PD-L1), and cytotoxic T-lymphocyte-associated antigen-4 (CTLA-4). Yet the same widespread immune activation that drives their therapeutic benefit renders patients susceptible to immune-related adverse events (irAEs). Among these cardiovascular toxicities have emerged as a particularly critical concern, drawing growing attention within the evolving field of cardio-oncology. ICI-associated cardiotoxicity encompasses a broad clinical spectrum, ranging from myocarditis and pericarditis to arrhythmias, heart failure, and vascular complications. Of these, immune-mediated myocarditis is the most severe, carrying a disproportionately high mortality risk despite its relatively low incidence. Its pathophysiology is multifaceted, driven by dysregulated immune activation, myocardial inflammation, T-cell-mediated cytotoxicity, aberrant cytokine release, and molecular mimicry between tumor and cardiac antigens. Additional mechanisms—including B-cell activation, complement cascade signaling, and inflammasome pathway engagement—may further amplify myocardial injury and heighten arrhythmogenic susceptibility. In this review, we summarize current insights into the immunological mechanisms underlying ICI-induced cardiotoxicity and examined the expanding clinical spectrum of cardiovascular irAEs. We further discuss strategies for early detection, risk stratification, and clinical surveillance, encompassing biomarker monitoring and multimodal imaging. Emerging therapeutic strategies targeting implicated immune pathways are also being explored to mitigate cardiovascular complications without compromising anticancer efficacy. A deeper mechanistic understanding will be essential for advancing the prevention, diagnosis, and management of ICI-associated cardiovascular toxicity in oncology practice.

## Highlights

- Immune checkpoint inhibitors (ICIs) induce a spectrum of cardiovascular immune-related adverse events including myocarditis, arrhythmias, pericardial disease, and heart failure with immune-mediated myocarditis carrying the highest mortality risk.
- ICI-associated cardiotoxicity is driven by dysregulated immune activation, encompassing T-cell–mediated myocardial injury, cytokine-driven inflammation, and molecular mimicry between tumor and cardiac antigens.
- Complementary mechanisms including complement cascade activation, B-cell dysregulation, and inflammasome signaling further amplify myocardial injury and heighten arrhythmogenic susceptibility.
- Integrated strategies combining biomarker surveillance, multimodal cardiac imaging, and targeted immunomodulatory therapy are essential for early detection, risk stratification, and mitigation of ICI-related cardiovascular toxicity without compromising anticancer efficacy.

## 1 Introduction

### 1.1 Cardio-oncology and the emergence of immune-mediated cardiotoxicity

The emergence of immune checkpoint inhibitor (ICI) therapies has transformed modern oncology by harnessing the immune system to recognize and destroy malignant cells. Over the past decade, ICIs have rapidly expanded from experimental immunotherapies to first-line treatments for numerous cancers. Their growing clinical adoption reflects both their novel mechanism of action and their ability to produce durable responses against malignancies that were historically difficult to treat. As the indications for ICIs continue to broaden across tumor types, the global population of patients receiving these therapies is steadily increasing.

Immune checkpoint inhibitors function by targeting regulatory pathways that normally restrain immune activation. Under physiological conditions, immune checkpoint molecules such as cytotoxic T-lymphocyte–associated protein 4 (CTLA-4) and programmed cell death receptor-1 (PD-1) help maintain immune homeostasis by preventing excessive T-cell activation and autoimmunity (1, 2). Tumor cells exploit these pathways by expressing checkpoint ligands that suppress cytotoxic T-cell responses, thereby allowing malignant cells to evade immune surveillance. ICIs block these inhibitory signals, restoring T-cell activation and enabling immune-mediated destruction of tumor cells (3).

Several major classes of ICIs are currently used in clinical practice. CTLA-4 inhibitors, including ipilimumab and tremelimumab, were among the earliest approved agents. They are primarily used to treat advanced melanoma, non-small cell lung cancer (NSCLC), malignant mesthelioma, and renal cell carcinoma (4, 5). PD-1 inhibitors, including pembrolizumab, nivolumab, and cemiplimab, or PD-L1 inhibitors, including avelumab, atezolizumab, and durvalumab, have become widely utilized against NSCLC, melanoma, and breast cancer (6–8). Collectively, these agents represent a paradigm shift in cancer therapy by modulating immune regulation rather than directly targeting tumor cells (9).

In addition to these established classes, a new generation of immune checkpoint therapies is currently emerging. Novel checkpoint targets-including lymphocyte activation gene-3 (LAG-3), T-cell immunoglobulin and mucin-domain containing-3 (TIM-3), and T-cell immunoreceptor with Ig and ITIM domains (TIGIT) are being investigated in clinical trials and early clinical use (10, 11). These next-generation ICIs aim to overcome resistance to existing therapies and further enhance antitumor immune responses through combination strategies and multi-checkpoint blockade. The expanding landscape of checkpoint targets highlights the continued innovation within immuno-oncology and suggests that ICI use will continue to rise in the coming years (12).

Despite their clinical benefits, ICIs are associated with a unique spectrum of immune-related adverse events (irAEs) resulting from nonspecific immune activation against healthy tissues. Among these complications, immune-mediated cardiotoxicity has emerged as a rare but potentially life-threatening condition. ICI cardiotoxicity encompasses a range of cardiovascular disorders, including myocarditis, pericardial disease, heart failure, arrhythmias, and ischemic events (13). Although the overall incidence remains relatively low compared with other toxicities such as dermatologic toxicity (rash, pruritus), endocrine dysfunction (hypothyroidism, hyperthyroidism), gastrointestinal toxicity (diarrhea, colitis), hepatotoxicity (transaminitis), and fatigue (14). The mortality rate associated with ICI-induced myocarditis is notably high, making early recognition and mechanistic understanding critically important (15).

Recent analyses have further clarified the spectrum and magnitude of cardiovascular risk associated with immune checkpoint inhibitor (ICI) therapy. Large observational and meta-analytic studies estimate the overall incidence of cardiac immune-related adverse events to range from approximately 0.8%–1.3%, with myocarditis representing the most frequently reported manifestation, occurring in roughly 0.5%–0.72% of treated patients (16). Although relatively uncommon, ICI-associated myocarditis remains particularly concerning due to its high mortality rate, estimated at approximately 37.7% (17). As continued advancements in oncology increasingly incorporate ICI therapy into the standard of care, more patients will be on ICI therapy, and thus an increasing number of people will be at risk for cardiac irAEs (18).

The precise mechanisms underlying ICI-associated cardiotoxicity remain poorly understood. Current evidence suggests that enhanced T-cell activation may trigger immune-mediated injury to cardiomyocytes and other cardiac tissues. However, growing evidence also implicates additional inflammatory and immunologic pathways in the development of cardiac damage, including B-cell dysregulation and excessive complement activation. In this review, we provide a comprehensive examination of the current understanding of ICI-associated cardiotoxicity, focusing on its proposed etiologies and pathogenic mechanisms. Particular attention will be given to emerging evidence supporting complement-mediated pathways as a potential contributor to cardiac injury during ICI therapy. By integrating current clinical and mechanistic data, this work aims to highlight critical knowledge gaps and identify novel directions for research into the prevention and management of ICI-related cardiovascular complications.

## 2 Immunopathogenesis of ICI-induced cardiotoxicity

This section aims to explore the functions of immune checkpoint targets, including PD-1, PD-L1, and CTLA-4, within the cardiac environment.

### 2.1 Function of ICI-targets in cardiomyocytes

Immune checkpoint inhibitor (ICI) therapies are broadly divided into three primary categories: programmed death-ligand 1 (PD-L1) inhibitors, programmed death receptor 1 (PD-1) inhibitors, and cytotoxic T-lymphocyte–associated antigen-4 (CTLA-4) inhibitors. The following section examines the mechanism of immune checkpoint inhibition, the physiological role of these checkpoint proteins in cardiomyocytes, and the potential mechanisms of cardiomyocyte injury that arise following pharmacologic blockade of these pathways.

#### 2.1.1 PD-1/PD-L1 axis

Programmed cell death protein-1 (PD-1) is a type I transmembrane receptor belonging to the CD28 family that is expressed on activated T cells, natural killer (NK) cells, B cells, monocytes, and dendritic cells (19–21). In contrast, programmed death ligand-1 (PD-L1) is expressed across a broader range of cell types, including T-cells, tumor cells, dendritic cells, macrophages, and other tissue cells by attenuating proliferation and cytokine secretion of PD-1-expressing cells while also promoting apoptosis (22, 23). This pathway is essential for maintaining peripheral immune tolerance and preventing autoimmune responses, and many tumor cells exploit this regulatory mechanism by overexpressing PD-L1, thereby suppressing cytotoxic T-cell responses and evading immune surveillance (24–26). This immune evasion strategy has made the PD-1/PD-L1 signaling pathway an important therapeutic target in modern cancer immunotherapy.

#### 2.1.2 Speculated function in cardiomyocytes

PD-L1 is expressed on cardiomyocytes and acts as an important mediator in cardiomyocyte cell injury. The PD-1/PD-L1 axis maintains a complex balance of immune regulation where expression has both positive and negative effects. In general, the role of PD-1/PD-L1 has been characterized as a short-term regulator that can protect the heart from excess immune-driven damage (25, 27, 28). The primary general function of PD-L1 is to prevent autoimmunity of T-cells. This is mediated through PD-1 interactions with PD-L1/B7-H1 and PD-L2/B7-DC. Specific to the heart, the PD-1/PD-L1 axis limits prevent the infiltration of CD8^+^ T cells into the myocardium (28). Aberrant infiltration is the crux of many autoimmune cardiovascular complications, such as myocarditis and pericarditis.

PD-1 also functions in a synergistic fashion with lymphocyte activation gene 3 (LAG-3) to mediate autoimmunity. LAG-3 normally contributes to negative regulation of CD4^+^ T cells, CD8^+^ T cells, and plasmacytoid dendritic cells (DCs). PD-1 and LAG-3 are commonly co-expressed in CD8^+^ T cells, and their simultaneous activation results in synergistic regulation against autoimmunity (29). In studies examining doxorubicin-induced cardiomyopathy, ligation of PD-1/PD-L1 in cardiomyocytes and T-lymphocytes led to decreased IFN-γ expression. This effect is more prominent with CD4^+^ lymphocytes than with CD8^+^ and is likely due to CD4^+^ activation being primarily independent, whereas CD8^+^ cells are activated by CD4^+^ initiation (30). Through the downregulation of the IFN-γ pathway, dissociation of the PD-1/PD-L1 complex may have many downstream cardiovascular effects. Notably, IFN-γ is implicated in the upregulation of atherosclerosis and cardiac fibrosis (31, 32).

Additionally, PD-L1 is coexpressed in a cardioprotective role in apoptotic and tissue remodeling pathways. Through the NF-κB signaling pathway, PD-L1 is expressed alongside caspase-3 as a key checkpoint molecule to control inflammation, which allows for proper tissue repair. Moreover, PD-1/PD-L1 attenuates the aberrant T-lymphocyte activation that impairs cardiac regeneration in neonatal cardiac injury (33). PD-L1 may contribute to cardiac repair through the modulation of the CD47/SHP2/SIRPα/SYK/FcγR pathway, resulting in better improvement of left ventricular function after myocardial infarction. This includes a downregulation of CD47/SHP2/SIRPα/SYK while FcγR is upregulated. The ultimate effect of these changes inhibits cGAMP production. cGamp is a notable mediator in exacerbating the severity of ischemia-reperfusion injury (34).

#### 2.1.3 CTLA4 axis

Cytotoxic T-lymphocyte-associated protein 4 (CTLA-4) is a surface cell receptor that is part of the CD28 immunoglobulin subfamily expressed primarily on T-lymphocytes. Through the interaction with its ligands, CD80 and CD86, CTLA-4 functions as an immune checkpoint inhibitor with a cell-intrinsic and a more predominant cell-extrinsic pathway (35–37). In the cell-extrinsic pathway, CTLA-4 is expressed by regulatory and conventional T cells (Treg, Tconv) to remove CD80/CD86 ligands on antigen-presenting cells (APCs) thereby suppressing the immune response (38). The intrinsic pathway involves the direct effects of CTLA-4 in T-cell by affecting T cell motility (36, 37).

#### 2.1.4 Speculated function in cardiomyocytes

CTLA-4-Ig has displayed cardioprotective therapeutic effects by preserving ejection fraction post-MI. T cell activation post-MI contributes to significant cardiac damage and reperfusion injury. Although the precise mechanism of action is not fully understood, there is evidence that CD28:CD80/86-mediated co-stimulation plays a role in the immune response during reperfusion. Thus, CTLA-4 can modulate the immune response and attenuate these effects by preventing interactions with CD28:CD80/86. Interestingly, this study also found that inflammation was a key contributor associated with reduced function independent of the amount of fibrosis (39).

CTLA-4 also plays an essential role in maintaining FOXP3^+^ Treg cells. FOXP3^+^ Treg cells are essential for maintaining T cell tolerance and have systemic effects throughout the entire body, including the myocardium and blood vessels (40). CD4^+^FoxP3^+^CD73^+^ Treg cells play an essential role in mediating inflammation resolution and cardiac healing post-MI. FoxP3^+^ Tregs in particular show a marked increase in IL2c, which has been demonstrated to have many therapeutic functions in attenuating heart failure progression. This is likely due to the suppression of effector T-cell mediated inflammation and modulation of fibroblasts (41).

In addition to the role of CTLA-4 on Treg cells, CTLA-4 also heavily influences CD4^+^ and CD8^+^ cell behavior. CTLA-4 in particular has important interactions with interleukin-12 (IL-12) in cytotoxic (CD8^+^) T-lymphocyte differentiation and function. Typically, IL-12 is essential for effective cytotoxic lymphocyte (CTL) differentiation. However, there is evidence that CTLA-4 ablated CTL can overcome a lack of IL-12 by proliferating in the cardiac-draining lymph node (CDLN). Even so, these cells demonstrated impaired cytotoxic functions and were unable to cause lethal myocarditis (42). Thus, this demonstrates that the proliferation of CD8^+^ T cells is influenced heavily by CTLA-4, and their cytotoxic function is more attributed to IL-12 signaling.

### 2.2 ICI-related B-cell dysregulation and cardiotoxicity

Although the primary effects of ICI therapy are largely mediated by T cells, there is growing evidence that checkpoint blockade also impacts B-cell tolerance. While much of the available data remains largely correlational, it highlights another potential mechanistic pathway that warrants further investigation. In the study by Das et al., combination therapy was associated with decreased circulating B cells, increased CD21^low^ B cells, and increased plasmablasts. Moreover, an early decline in B-cell numbers was associated with both earlier onset of toxicity and greater severity of immune-related adverse events (IRAEs). Notably, these findings appeared to be specific to B-cell changes and were not observed in circulating T-cell, NK-cell, or myeloid-cell populations (43).

As of yet, it remains unclear whether the effects on B cells occur indirectly through T-cell–mediated mechanisms or directly through signaling involving B-cell receptors. CD21^low^ B cells were also shown to express PD-1 receptors, making them a potential direct target of checkpoint inhibitors (43). CD21^low^ B cells have been implicated in several autoimmune diseases, including systemic lupus erythematosus (SLE), rheumatoid arthritis (RA), common variable immunodeficiency (CVID), and Crohn's disease (44). An alternative, more indirect mechanism may occur through interactions between CD21^low^ B cells and T-follicular helper or other T-helper cell subsets (45).

Although CD21^low^ B cells themselves have not been directly linked to cardiac effects, there is some evidence suggesting a potential association between elevated CD21^low^ levels and increased pulmonary arterial pressure as well as higher renal resistive index in patients with systemic sclerosis, compared with patients who have lower CD21^low^ levels (46). In addition, CD21^low^ B cells in general are implicated in several cardiovascular pathways including cardiac homeostasis, remodeling, and fibrosis. Firstly, cardiac B cells maintain inflammatory homeostasis with T cells, macrophages, endothelial cells, monocytes, and fibroblasts through transforming growth factor beta-1 (TGF-β1) and IL-10. Moreover, mature B cells are often recruited in response to cardiac injury post-MI or in heart failure. The effects of B cell activity in the heart are complex and multifactorial (47). For instance, IL-10–producing B cells in pericardial adipose tissues reduces cardiac remodeling following acute MI (48). In other cases, B cells may increase pathogenic cardiac remodeling via inflammatory cytokines like IL-1β and IL-6 (49). Collectively, current evidence suggests that checkpoint blockade induces significant perturbations in B-cell homeostasis, particularly expansion of CD21^low^ B-cell populations associated with autoimmune phenotypes. However, the majority of supporting data are translational and correlational, with limited direct evidence linking these cells to myocardial injury. Future studies incorporating myocardial tissue analysis, longitudinal immune profiling, and B-cell-targeted therapeutic interventions will be required to determine whether B-cell dysregulation represents a causal contributor to ICI-myocarditis or simply a biomarker of systemic immune activation.

### 2.3 Complement-mediated cardiotoxicity

Complement activation has emerged as a potential contributor to immune-related adverse events (irAEs) associated with immune checkpoint inhibitor (ICI) therapy. Although ICIs primarily modulate T-cell–mediated immune responses, several case reports and mechanistic studies suggest that dysregulation of the complement system may also play a role in the development of immune-mediated toxicity. In multiple reported cases of ICI-induced complications, complement activity was markedly elevated, and clinical improvement was observed following treatment with complement inhibitors, suggesting a causal role for complement-mediated inflammation. One proposed mechanism involves off-target effects of therapeutic monoclonal antibodies that trigger complement activation through antibody-mediated immune responses. For example, in ICI-induced hypophysitis associated with CTLA-4 blockade, a type II hypersensitivity reaction framework has been proposed. In this model, anti–CTLA-4 antibodies bind CTLA-4 expressed on pituitary endocrine cells, leading to activation of the classical complement cascade and deposition of complement components such as C3d and C4d, ultimately resulting in inflammatory tissue injury (50). These findings demonstrate that ICIs may induce complement-mediated cytotoxicity through antibody-dependent mechanisms in susceptible tissues. Thus, the precise contribution of complement pathway activation to ICI-induced myocarditis is still largely inferential, drawn from mechanistic parallels with viral myocarditis, dilated cardiomyopathy, and non-cardiac irAEs such as hypophysitis and thrombotic microangiopathy. Direct evidence linking complement dysregulation to human ICI-associated cardiac injury is limited to case reports and indirect mechanistic data; prospective studies measuring complement component levels, including C3a, C5a, C3d, and C4d deposition, in patients developing cardiac irAEs are conspicuously absent. Addressing this gap is essential to determine whether complement inhibition represents a viable therapeutic strategy in this context.

Complement dysregulation has also been implicated in other ICI-associated autoimmune conditions. Experimental work examining ICI-triggered sialadenitis demonstrated that anti–PD-1 therapy can induce complement activation within salivary gland tissue, leading to early glandular injury through complement-dependent cytotoxicity (51). Similarly, case reports have described complement-mediated thrombotic microangiopathy (TMA) developing during ICI therapy, in which reduced complement levels and clinical improvement with complement pathway inhibition suggest that checkpoint blockade may provoke autoantibody formation against complement regulatory factors (52). Although direct evidence linking complement activation to ICI-induced cardiotoxicity remains limited, several lines of evidence suggest a plausible mechanistic connection. Complement-mediated injury has long been implicated in cardiac pathologies such as dilated cardiomyopathy (DCM), where antibody-dependent complement activation can lead to cardiomyocyte damage and progressive ventricular dysfunction (53). In addition, dysregulated complement activation has been observed in viral myocarditis, where pathogens such as coxsackievirus B3 trigger immune-mediated myocardial injury and contribute to the development of heart failure. Given the established role of complement in immune-mediated cardiomyocyte injury, similar pathways may plausibly contribute to cardiac inflammation and tissue damage observed in ICI-associated myocarditis (54).

Notably, C3 and C3a serve as a strong chemotactic to draw monocytes to ischemic areas. Subsequent complement activation results in cell death of ischemic tissue, cardiac fibrosis, and heart dysfunction. Moreover, inhibition of C3 has been demonstrated to reduce myocardial necrosis post-ischemia (55). Complement C3a des-Arg or acylation-stimulating protein (ASP) is produced after C3 cleavage and rapid conversion of C3a to ASP by carboxypeptidases B and N (56). Although predominantly implicated in insulin resistance, ASP is activated in the human heart and is associated with cardiac dysfunction as a potential predictor of heart failure progression (57).

**Figure 1:**
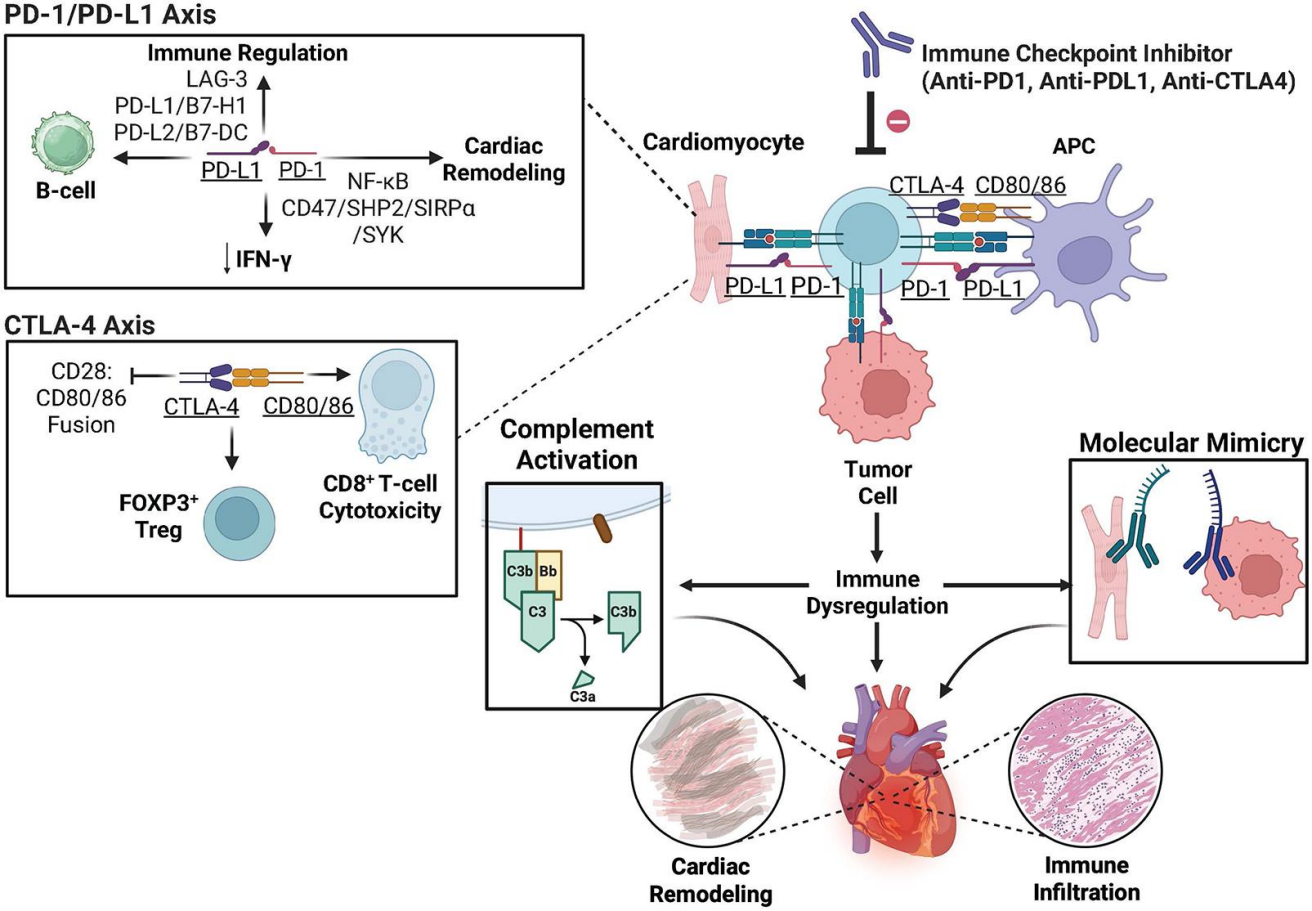
Proposed immunophysiologic mechanism of ICI targets in cardiomyocytes. This schematic illustrates native role of the many immune checkpoint inhibitor (ICI) targets in the cardiomyocytes and the subsequent effects by which ICI therapy dysregulates the native cardioprotective roles. The PD-1/PD-L1 Axis provides a cardioprotective function in mediating cardiac remodeling via the NF-κB and CD47/SHP2/SIRPα/SYK/FcγR pathway; reduction of the pro-inflammatory cytokine IFN-γ; immune regulation via LAG-3, PD-L1/B7-H1, and PD-L2/B7-DC; and regulation of B-cell tolerance. The CLTA-4 Axis provides cardioprotective effects by mediating T-cell tolerance through the Foxp3^+^ Treg while also modulating the cytotoxicity of CD8^+^ T-cells. Upon immune dysregulation by the aforementioned mechanisms, the immune response is susceptible to be exacerbated by other mechanisms like complement activation and molecular mimicry which furthers adverse cardiac remodeling/fibrosis as well as aberrant immune infiltration. [Made using Biorender.com].

In response to cellular stress, toll-like receptor (TLR) 2/4 stimulation by heat shock protein 60 (HSP60) and lipopolysaccharide (LPS) upregulated the Ca2+/Calmodulin-dependent kinase II (CaMKII) (58). The CaMKII is an essential proinflammatory regulator of myocardial inflammation via C3 and Complement Factor B activation in cardiomyocytes (59). CaMKII may aggravate injury and fibrosis of the myocardium, leading to structural damage and dysfunctional myocardium. This has brought CaMKII into the spotlight as a potential therapeutic target for several cardiovascular disorders (60). Interestingly, CaMKII shares several overlapping pathways with PD-1 and CTLA-4, such as the NFAT signaling pathway (61–63). Thus, CaMKII interactions with ICI therapies may be areas of future studies. Taken together, these findings suggest that complement activation may represent an underrecognized immunologic pathway involved in ICI-related toxicity. While current evidence remains largely indirect and based on case reports or mechanistic parallels with other diseases, further investigation into complement signaling during checkpoint blockade may reveal novel therapeutic targets and improve the management of severe immune-related adverse events. Figure 1 summarises the pathophysiological drivers of ICI-associated cardiac injury and cross-reactive molecular mimicry that manifest histologically as severe cardiac remodeling and immune infiltration. Table 1 summarizes mechanisms with level of evidence avaible in the literatures as established or emerging based on preclinical, translational, and clinical research.

**Table 1:** Mechanisms implicated in ICI-cardiotoxicity.

**Mechanism**	**Evidence Level**	**Supporting Evidence**
CD8+ T-cell myocardial injury	Established	Human ICI-myocarditis biopsy studies (212, 213), translational studies (98), murine depletion models (42, 97, 214).
Cytokine dysregulation (IL-6, TNF, IFN-γ)	Established	Human ICI-myocarditis biopsy studies (65, 215, 216), translational studies (217), murine depletion models (218, 219).
Treg dysfunction/loss of tolerance	Established-Emerging	Murine depletion models (40, 220), mechanistic link (37, 41), evidence of Treg dysfunction in ICI-patients (221).
Macrophage-T cell inflammatory loops	Established–Emerging	Human ICI-myocarditis biopsy studies (65, 222) and preclinical studies (223), Murine depletion models (100).
B-cell dysregulation/CD21^low^ expansion	Emerging	Human ICI-myocarditis biopsy studies (67, 77, 224), autoimmune disease parallels (225, 226), Murine depletion models (107). Mechanistic support in other forms of myocarditis (106, 227).
Inflammasome/NLRP3 signaling	Emerging	Predominantly preclinical evidence (86).
Complement-mediated cardiotoxicity	Hypothetical	Case reports (104, 213, 228), mechanistic extrapolation from other irAEs (51, 52, 209), and myocarditis models (54, 59).
Humoral autoantibody-mediated injury	Hypothetical	Limited human evidence, indirect mechanistic support (66).

The table details the discussed mechanisms, classifying their level of evidence as established or emerging based on preclinical, translational, and clinical research.

### 2.4 Cancer microenvironments that modulate susceptibility to cardiotoxicity

Frequency of cardiac irAEs varies across patients with melanoma, lung cancer, and kidney cancer receiving dual immune checkpoint inhibitors (ICIs). Disproportionality analyses revealed that kidney cancer patients exhibited the highest reporting odds ratios (RORs) for myocarditis and atrial fibrillation, whereas lung cancer patients showed the strongest signals for cardiac failure and pericardial effusion, while melanoma patients demonstrated comparatively lower signals for cardiac failure, highlighting tumor-specific differences in the clinical spectrum and risk of ICI-associated cardiotoxicity (64). Although these findings may be influenced by inherent cardiotoxic effects of the tumor, there may be additional underlying mechanisms of certain tumors that predispose to ICI-induced cardiotoxicity.

Molecular mimicry between the tumor microenvironment and cardiomyocytes may be a potential mechanism for cardiotoxicity. RNA sequencing of melanoma tumors has revealed expression of cardiac troponin and myosin heavy chain genes, and TCR sequencing demonstrated identical T cell clones infiltrating the tumor, skeletal muscle, and inflamed myocardium (65). The α-Myosin heavy chains have also been demonstrated to be a key autoantigen in murine models because this protein may escape central tolerance due to absence from the thymus. Moreover, this process seems to be mediated by MHC class I complexes (66). With that being said, there is growing evidence now that this hypothesis may be more complex than previously thought. In a study by Blum et al. across fifty-two heart-expanded T-cell receptor clones, TCRs enriched in heart tissue did not recognize the putative cardiac autoantigens α-myosin, troponin I, or troponin T in immune-related myocarditis. This demonstrates that there may be an additional unidentified antigen involved in disease pathogenesis (67).

In addition to mimicry, tumors may also generate a baseline autoimmune environment that predisposes to cardiotoxicity. Thymomas or thymic epithelial tumors (TETs) are rare mediastinal neoplasms originating from thymic epithelial cells (68, 69). Current therapies for recurrent tumors have low response rates, and therefore, ICI therapies have been a potential alternative treatment for high-grade, unresectable cases. However, treatment of TETs is associated with a high risk for irAEs, including myocarditis (70–73). These cancers inherently disrupt AIRE/FEZF2-driven tissue-restricted antigen expression and impair regulatory T cell generation, permitting escape of autoreactive T cells (74). Thus, TETs are associated with a high baseline rate of paraneoplastic autoimmune syndromes such as myasthenia gravis (75). More importantly, it is demonstrated that myocarditis occurs earlier in ICI-treated patients who are positive for anti-acetylcholine receptor antibodies and have thymic epithelial tumors (TET). These individuals are more susceptible to life-threatening arrhythmias, concurrent myositis, severe respiratory muscle failure, and death (76, 77). Similar traits are shared by other malignancies such as small cell and squamous cell lung cancer, melanoma, and kidney cancer (78–80).

## 3 Clinical spectrum of ICI-associated cardiac toxicities

Immune checkpoint inhibitor (ICI) therapy is increasingly recognized to carry a meaningful risk of cardiovascular toxicity, encompassing a broad and clinically significant spectrum of adverse events (irAEs). Among these, myocarditis emerges as the most frequently reported and one of the most severe complications, characterized by high mortality rates. Other commonly observed cardiac toxicities include atrial fibrillation, heart failure, pericardial effusion, and myocardial infarction, each contributing variably to morbidity and mortality (64). The following sections will explore each of these cardiac irAEs in terms of prevalence, clinical presentation, and underlying immunological mechanism.

### 3.1 Myocarditis and pericarditis

Myocarditis and pericarditis is life-threatening irAE associated with significant morbidity and mortality and therefore warrants early recognition and prompt management. The 2025 European Society of Cardiology (ESC) Guidelines on Inflammatory Myocardial and Pericardial Syndromes (IMPS) introduce a unified conceptual framework that integrates myocarditis, pericarditis, and overlapping entities such as myopericarditis and perimyocarditis into a single spectrum of inflammatory cardiac disease. Thus, the following section will examine the implications of ICI therapy on the spectrum of inflammation across the myocardium and pericardium. This framework acknowledges that myocardial and pericardial inflammation frequently coexist and may represent different manifestations of a shared immune-mediated pathophysiologic process (81).

Patients commonly present with nonspecific symptoms such as fatigue, dyspnea, chest pain, palpitations, or signs of heart failure, which can complicate early diagnosis. The onset of ICI-associated myocarditis is highly variable but typically occurs early during treatment in the first one to two months of therapy initiation (70). Although myocarditis is increasingly recognized in patients receiving immune checkpoint inhibitors, clinically significant cases remain relatively rare compared to other immune-related adverse events, with an estimated incidence of 0.5%–1.2% (82). Clinical presentation varies widely with symptoms including chest pain, dyspnea, unexplained arrhythmias, heart failure, and cardiogenic shock (83). Despite its low incidence, the condition carries a poor prognosis, with reported mortality rates between 25% and 50%. Notably, the risk appears to be higher in patients receiving combination checkpoint inhibitor therapy (82).

**Figure 2:**
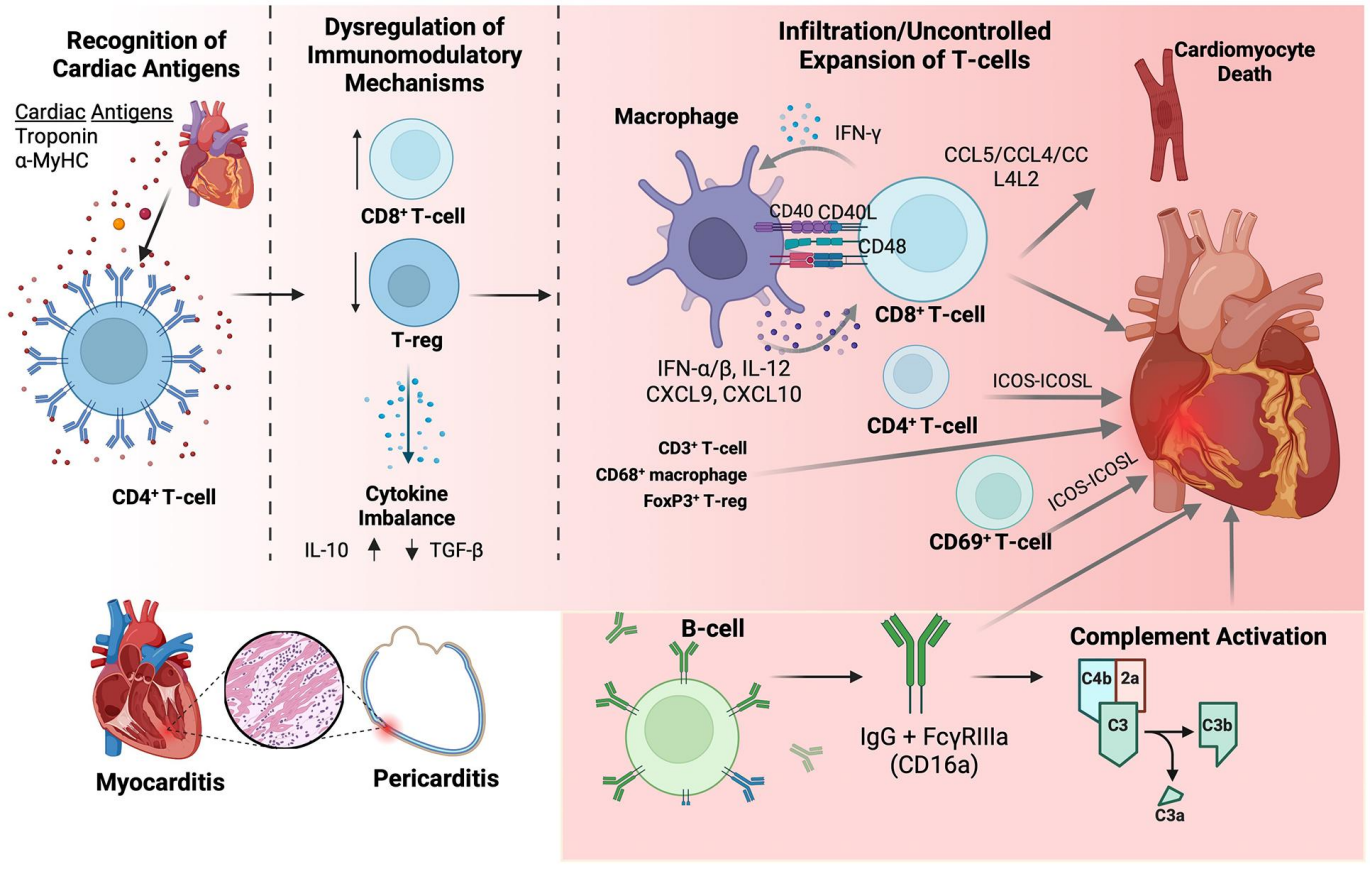
Mechanisms of ICI-induced myocarditis. The schematic provides a sequential mechanism by which ICI-therapy results in inflammatory myocardial and pericardial diseases. This begins with CD4^+^ T-cell recognition of cardiac antigens like troponin and α-Myosin Heavy Chain (α-MyHC). Subsequent dysregulation of the immune system by ICI-therapies involves a decrease in the amount of T-regs, increase in CD8^+^ T-cell expression, and pro-inflammatory cytokine imbalance as IL-6 increases and TGF-β decreases. Afterwards, the stage is set for numerous pathways of lymphocyte and mononuclear infiltration including CD3+, CD4+, and CD8^+^ T cell, CD68^+^ macrophages, and FoxP3^+^ T cells. In particular, CD8+ T-cell infiltration is further amplified by a positive feedback loop with CCR2^+^ macrophages with the IL-12b, TNF, and IFN-γ signaling pathway as well as the CXCL9/10 pathway. Moreover, CXCR3 receptor expression and upregulation of the CCL5/CCL4/CCL4L2 pathway can promote direct cardiomyocyte death. CD4+ and CD69+ T-cell infiltration is mediated by upregulation of the ICOS-ICOSL signaling pathway. Lastly, B-cells may mediate autoimmune myocarditis through the secretion of IgG against cardiac myeloid cells that have the receptor FcγRIIIa (CD16a). This leads to subsequent complement activation that exacerbates myocarditis. [Made using Biorender.com].

In addition to myocarditis, other inflammatory cardiac syndromes such as pericarditis and myopericarditis have also been increasingly reported in patients receiving checkpoint blockade. A large retrospective study using the TriNetX database evaluated outcomes in patients diagnosed with pericarditis, myocarditis, or myopericarditis within one year of initiating ICI therapy. The investigators identified 3,661 patients with ICI-associated pericarditis, 226 with myocarditis, and 46 with myopericarditis, and compared outcomes with matched non-ICI populations. Across all three conditions, ICI-associated disease was associated with significantly increased mortality. One- and five-year mortality rates were higher among patients with ICI-related pericarditis (44.3% vs. 37.3% and 54.3% vs. 46.9%), myocarditis (38.9% vs. 20.8% and 47.8% vs. 34.3%), and myopericarditis (56.5% vs. 28.3% and 63.0% vs. 28.3%). Multivariable analysis demonstrated a significantly elevated risk of mortality across multiple time points, with hazard ratios of 1.82 at 90 days and 2.04 at one year for myocarditis. These findings highlight the substantial clinical impact of ICI-associated cardiac inflammation and underscore the need for improved surveillance and management strategies (84). Altogether, the following section will explore the established and hypothesized mechanisms that underlie aberrant ICI-induced inflammation across the spectrum of the myocardium and pericardium. These mechanisms are shown together in Figure 2.

#### 3.1.1 T-cell and macrophage mediated injury

The pathogenesis of immune checkpoint inhibitor (ICI)–induced myocarditis is most consistently characterized by a dysregulated, T cell–driven immune response that culminates in myocardial inflammation and injury. Under physiologic conditions, inhibitory pathways such as PD-1/PD-L1 and CTLA-4 maintain peripheral tolerance and prevent autoreactive T-cell activation. Pharmacologic blockade of these checkpoints enhances antitumor immunity but simultaneously lowers the threshold for autoimmune responses, including those targeting cardiac tissue (85). However, the process by which this autoimmune response leads to myocardial and pericardial infiltration can be broken into several key steps at which ICI-therapies interfere. These are as follows: 1) recognition of cardiac antigens by circulating T-cells, 2) dysregulation of crucial immunomodulatory mechanisms, and 3) infiltration and uncontrolled expansion of T-cells resulting in myocarditis and pericarditis.

1. *Recognition of Cardiac Antigens:* CD4^+^ most predominantly targets cardiac proteins such as troponin I (TnI) and myosin. The mechanism of this begins first by the release of cardiomyocyte-derived protein antigens due to tissue damage. These antigens are then sensed by dendritic cells (DCs) via TLR-mediated signaling. Subsequently, these TLRs interact with the myeloid differentiation primary response 88 through an immunoproteasome, like LMP7, to affect changes in the T-cell effector pathway (88).
  Other studies have also implicated cardiac α-myosin heavy chain (α-MyHC) as a key target for autoreactive T-cells. α-MyHC is a contractile protein expressed in cardiac muscle. The α-MyHC has been demonstrated to be an autoantigen in mouse models on MHC-II restricted models (87). Moreover, α-MyHC may share structure with tumor antigens, and it has been hypothesized that molecular mimicry may result in cross-reactivity between anti-tumor cytotoxic T cells and α-MyHC expressed in the heart (88, 89).
2. *Dysregulation of crucial immunomodulatory mechanisms:* To begin, ICI therapies have been found to decrease the number of Tregs. This was accompanied by increased inflammatory cell infiltration and elevated CD8⁺ T-cell expression, suggesting loss of immune tolerance and enhanced effector T-cell activation within the myocardium (21). Tregs play a central role in maintaining immune homeostasis by suppressing excessive T-cell responses, limiting autoreactive lymphocyte activation, and preventing tissue-specific autoimmune injury (90). Via Treg dysregulation, PD-1 inhibition can thus weaken negative immune regulation, allowing for unregulated lymphocyte activation and autoimmune side effects such as myocarditis.
  The functional consequences of Treg depletion are further reflected in cytokine imbalance and amplified inflammatory signaling. Tregs normally exert immunosuppressive effects through cytokines such as IL-10 and TGF-β, which inhibit effector T-cell proliferation, regulate antigen-presenting cell activity, and promote peripheral tolerance (91). In this model, increased IL-6 levels likely contributed to reduced Treg differentiation, as IL-6 antagonizes TGF-β–induced Treg development while promoting Th17 polarization, thereby shifting the immune response toward inflammation. Although IL-10 levels increased, potentially as a compensatory response, reduced Treg numbers may limit its regulatory effectiveness in controlling autoimmune damage (21). TGF-β, produced by multiple immune cell populations, normally suppresses CD4⁺ T-cell proliferation by inhibiting IL-2 production and cell-cycle progression while inducing regulatory T-cell subsets such as Th3 cells (92). The altered TGF-β expression observed in myocardial tissues also corresponded with disease severity, suggesting impaired Treg-mediated regulation (21). Collectively, these findings indicate that PD-1 inhibition disrupts Treg differentiation and function, leading to diminished immune suppression, cytokine imbalance, and enhanced inflammatory injury in autoimmune myocarditis.
3. *Infiltration and uncontrolled expansion of T-cells in Myocardial and Pericardial Tissue:* ICI therapy that leads to irAEs typically results in a significant increase in lymphocyte proliferation and mononuclear cell infiltration (93). Specific to ICI-related myocarditis, the predominant infiltrates are CD3^+^, CD4^+^, and CD8^+^ T lymphocytes, CD68^+^ macrophages, and FoxP3^+^ T regulatory cells. Among the listed populations, CD8+ and CD4+ T cells represent the predominant cell subsets (94).
  CD8^+^ T cells in particular play a direct role in myocardial injury and in the induction of myocarditis. In a murine model, the clonally expanded cardiac immune infiltrate predominantly consisted of CD8⁺ T cells. Interestingly, selective depletion of CD8⁺–not CD4⁺-T cells rescued survival and prevented disease (28, 65, 95, 96). Furthermore, adoptive transfer experiments confirmed that CD8⁺ T cells are the major driver of myocarditis, likely dependent on CD8T cell-derived tumor necrosis factor (TNF) and TNF receptor 2 (TNFR2) signaling pathways (97). Moreover, α-myosin acts as an MHC-I-restricted autoantigen and is recognized by the antigen-specific cytotoxic CD8⁺ T cell, which initiates the pathogenesis of ICI-induced myocarditis (66).

Furthermore, CD45RA^+^ re-expressing effector memory CD8^+^ T cells (TEMRA) were found to show the most clonal expansion upon activation during ICI-myocarditis. In particular, these cells highly express the CXCR3 receptor, which further promotes lymphocyte chemotaxis to areas of inflamed tissue like the heart. Additionally, these cardiac effector memory CD8^+^ cells also upregulated CCL5/CCL4/CCL4L2 (98). The aforementioned upregulated CD8^+^ T cells also directly contribute to cardiomyocyte death through increased expression of cytolytic mediators, including granzymes (A, B, and K), perforin, and Fas ligand. This is accomplished via both perforin/granzyme-mediated apoptosis and Fas–FasL signaling pathways (88, 94).

A positive feedback loop has been identified between CCR2⁺ macrophages and CD8⁺ T cells that amplifies myocardial inflammation through coordinated IL-12b, TNF, and IFN-γ signaling pathways (99). CCR2⁺ macrophages promote activation and expansion of cytotoxic CD8⁺ T cells, which in turn release pro-inflammatory cytokines that further stimulate macrophage recruitment and activation, thereby sustaining and intensifying cardiac immune responses. This reciprocal signaling network contributes to persistent inflammatory injury within the myocardium. Furthermore, prior exposure to CD40 agonists was shown to sensitize cardiac tissue to subsequent inflammatory insults, accelerating pathologic left ventricular remodeling and exacerbating disease progression, suggesting that earlier immune activation events can prime the heart for heightened inflammatory susceptibility (99).

Another observed positive inflammatory feedback loop occurs from activated macrophages secreting CXCL9 and CXCL10. This attracts CXCR3-expressing effector T cells to cardiac tissues and further exacerbates cardiac inflammation. Additional inflammatory mediators, including interleukins and signaling pathways such as NF-κB (via IKK activation), further contribute to the pro-inflammatory milieu (100, 101). This creates a self-perpetuating positive feedback loop, leading to progressive immune cell infiltration and sustained myocardial inflammation.

Additionally, preclinical models demonstrate increased populations of activated CD69^+^ T cells alongside upregulation of T-cell receptor signaling, CD28 co-stimulation, and inducible T-cell co-stimulator (ICOS–ICOSL) pathways (102). CD4^+^ T cells have also been shown to lead to ICI-induced myocarditis through the inducible T-cell co-stimulator (ICOS) pathway. ICOS was significantly expressed on the majority of infiltrating CD4^+^ cells of the myocardium (102). These findings suggest persistent antigen presentation and sustained T-cell activation within cardiac tissue. Collectively, the results of these studies support a model in which ICI therapy disrupts immune tolerance, leading to enhanced antigen presentation, robust activation of cytotoxic and helper T-cell subsets, and macrophage-mediated amplification of inflammation. The resulting immune cascade drives cardiomyocyte injury and underlies the clinical manifestations of ICI-associated myocarditis.

#### 3.1.2 B-cell and antibody mediated activation

Although ICI-myocarditis is largely T-cell and macrophage mediated, there is growing evidence that B-cell and humoral activation may also be influenced by ICI therapies and thus may factor into cardiac irAEs. One potential hypothesis is that structural cardiomyocyte damage, through surgical resection or chemoradiotherapy, may result in cardiac-specific autoantigen release alongside activation of autoreactive B cells. This would subsequently lead to the development of myocardial necrosis and inflammation (88, 97). These findings are well studied in other pathologies of myocarditis, like that of coxsackievirus (CVB3), where B cells were even observed directly infiltrating the heart and leading to antibody-dependent enhancement of CVB3 entry into the myocardium. Moreover, B cells can increase myocardial inflammation through interactions with and downregulation of T cells (103).

In the study published by Siddiqui et al, B cell response was implicated in patients with myocarditis. The B cell response led to the secretion of IgG engaging with FcγRIIIa (CD16a) on C1qhiMRC1^+^ myeloid cells, leading to downstream complement activation. This observation has spurred on currently ongoing B cell intervention strategies for myocarditis (104). Other studies have demonstrated that the frequency of serum markers for B cells and plasma cells were decreased in patients with ICI-induced myocarditis, bringing into question the significance of B lymphocytes (105). Thus far, it is too early to definitively determine whether or not B-cells have a significant role.

Although aberrant complement activation is not directly implicated in ICI-induced myocarditis, there are several prospective mechanisms for aberrant complement activation to affect myocarditis. Complement dysregulation has become a novel mechanism for viral myocarditis. Coxsackievirus B3 specifically produces proteinase that cleaves the complement proteins CD59/protectin and CD55/DAF. These complement proteins play an essential role in preventing auto injury to the heart (70). Moreover, complement serves as an important factor in initiating autoimmune myocarditis. Early depletion of C3 alongside a blockade of complement receptor type 1 (CR1) and type 2 (CR2) is associated with a reduction of proinflammatory cytokines such as TNF-α and IL-1, as well as reduced production of cardiac myosin–specific autoantibody. Moreover, CR1 and CR2 may also play a role in B and T cell activation (106). In several preliminary studies, ICI therapies have been associated with the release of cardiac damage-associated molecular patterns (DAMPs) as a result of off-target interactions with the myocardium. These include vascular expression of NF-kB, systemic SDF-1, IL-1β, IL-6 levels, and myocardial NLRP3 (107). Although the data are still preliminary, the implications of myocardial DAMP release from ICI may also trigger complement activation through anaphylatoxins C3a and C5a, resulting in potential cardiomyocyte damage (108). The key questions that remain unanswered are whether complement activation is a direct mediator of myocardial injury or merely a downstream consequence of tissue damage, if complement inhibition represents a viable therapeutic strategy in ICI-myocarditis, given the current absence of prospective clinical trial data, and whether humoral immune mechanisms identify a distinct subset of ICI-myocarditis patients who may benefit from B-cell–targeted therapies such as anti-CD20 agents (109–111). Figure 2 illustrates stepwise pathophysiological cascade of immune checkpoint inhibitor (ICI)-induced cardiotoxicity.

### 3.2 Atherosclerosis and vasculitis

#### 3.2.1 Atherosclerosis

Atherosclerotic cardiovascular disease (ASCVD) refers to a spectrum of conditions caused by the buildup of lipid-rich atherosclerotic plaques within arterial walls, leading to progressive narrowing or sudden occlusion of blood vessels. Clinical manifestations include myocardial infarction, ischemic stroke, and the need for coronary revascularization, all of which arise from plaque rupture or impaired arterial blood flow. ASCVD represents a leading cause of morbidity and mortality worldwide and is traditionally driven by risk factors such as hyperlipidemia, hypertension, diabetes, smoking, and aging (112). More recently, chronic inflammation and immune system dysregulation have been recognized as central contributors to atherosclerosis development and progression. This understanding has raised concern that immune checkpoint inhibitors (ICIs), which enhance immune activation to treat malignancies, may unintentionally accelerate vascular inflammation and promote atherosclerotic disease.

Emerging epidemiologic evidence suggests an association between ICI therapy and increased ASCVD risk, although findings remain heterogeneous. In a large matched cohort study of 2,842 patients initiating immunotherapy compared with 2,842 age-matched controls, the two-year risk of myocardial infarction (*n* = 37, 1.30%), coronary revascularization (*n* = 22, 0.77%), or ischemic stroke (*n* = 35, 1.23%) was approximately threefold higher among patients receiving ICIs, accompanied by more than a threefold increase in plaque progression on imaging (113). Similarly, Bar et al. reported acute vascular events in 2.6% of 1,215 cancer patients treated with ICIs, including myocardial infarction in 0.66% within six months of therapy initiation (114). Collectively, these studies indicate a potential elevation in ASCVD risk associated with ICIs, while highlighting ongoing uncertainty regarding magnitude, causality, and patient-specific susceptibility. Immune checkpoint inhibitors (ICIs) enhance vascular inflammation by modulating immune responses involving T cells and macrophages, thereby contributing to the development and progression of atherosclerosis. Progression of atherosclerosis can be defined by the following steps: Endothelial dysfunction, atherosclerosis and fatty streak formation, fibrous plaque development, and finally plaque stability or rupture (115). Each of these steps are heavily mediated by inflammatory mechanism.

Atherosclerosis first begins with endothelial damage. The extent of endothelial damage is typically related to mechanical stress on the wall, which is related to hemodynamic changes such as blood pressure and the vessel architecture. Through mechanisms of inflammation and cytotoxicity, ICI-therapies may result in endothelial damage. Through the induction of intercellular adhesion molecule-1(ICAM-1) and inducible nitric oxide synthase (iNOS), the PD-1 inhibitor, Pembrolizumab, increases leukocyte infiltration, allowing for endothelial damage (116). Immune checkpoint inhibitors greatly increase T lymphocyte to macrophage ratio. For that reason, atherosclerotic processes are lymphocyte-dominant (117). Inhibition of immune checkpoint signaling promotes heightened immune activation and facilitates the recognition of antigens such as oxidized low-density lipoprotein (oxLDL) and heat shock proteins (HSPs) presented by APCs. These interactions stimulate T helper (Th) cell responses and increase the production of pro-inflammatory mediators, including interferon-γ (IFN-γ), tumor necrosis factor-α (TNF-α), and multiple interleukins (ILs) (28, 31, 85). Via these pro-inflammatory mechanisms, ICI-therapies create a pro-inflammatory, atherogenic environment. This also includes macrophage polarization to M1 macrophages activated through NF-κB and JAK/STAT signaling pathways, which are pro-inflammatory and atherogenic in contrast to their anti-inflammatory, M2 counterparts (118). The M1 macrophages consume the oxLDL to form lipid-laden macrophages known as foam cells. These foam cells are a major building block of the lipid-rich necrotic cores in plaques (119). In particular, C-X-C Motif Chemokine Ligand 9 (CXCL9) is a predominant cell surface marker that is correlated with M1 macrophage expression in the setting of ICIs (31, 118, 120).

**Figure 3:**
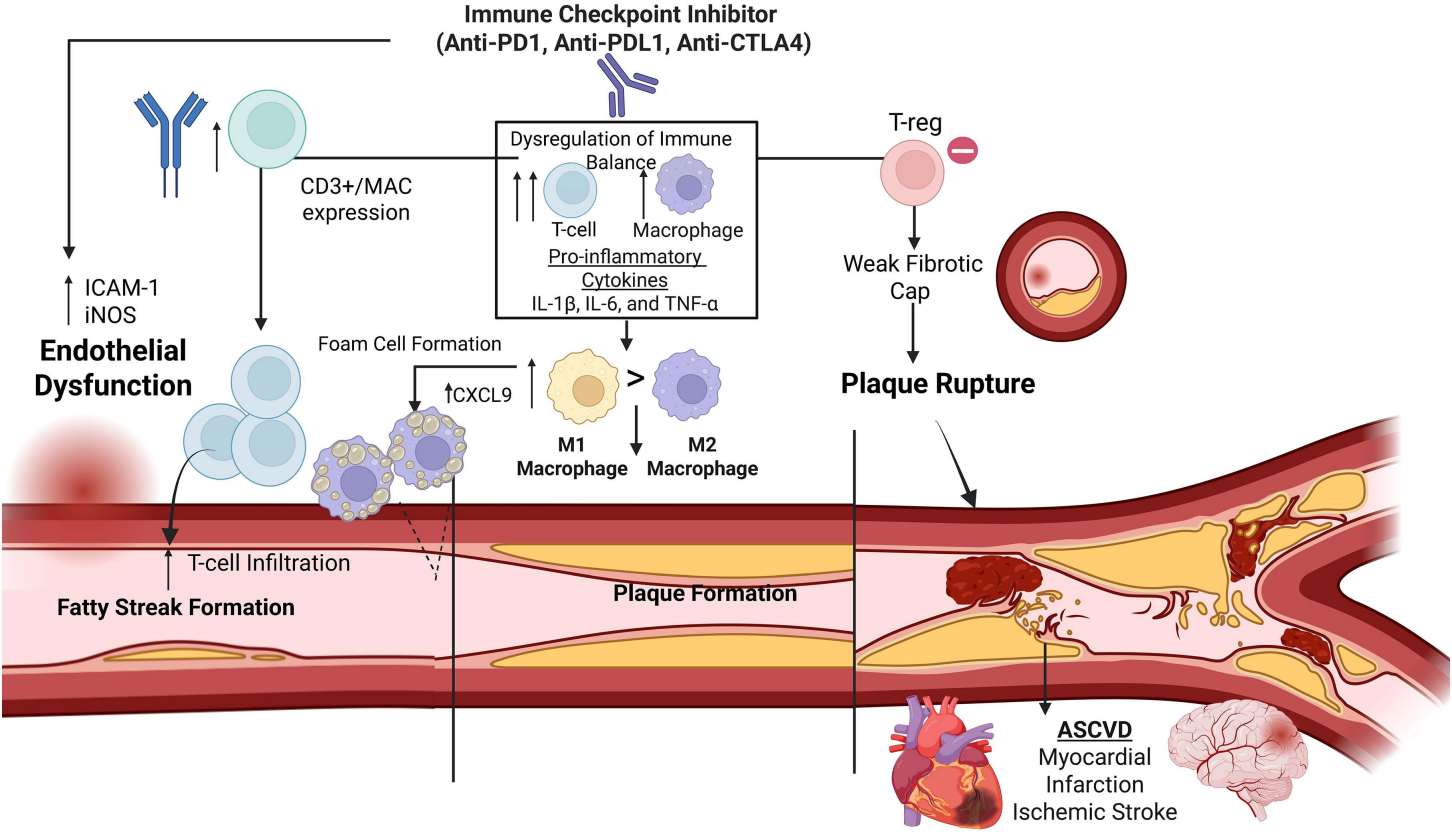
Mechanisms of ICI-induced atherosclerosis. This schematic provides an overview of the process of how ICI-therapies promote atherosclerotic plaque development and increase Atherosclerotic Cardiovascular Disease (ASCVD) risk for myocardial infarction and ischemic stroke. First, endothelial dysfunction, the initiation of atherosclerosis, is exacerbated by ICI-induced promotion of leukocyte infiltration through induction of intercellular adhesion molecule-1(ICAM-1) and inducible nitric oxide synthase (iNOS) upregulation. Secondly, the a pro-inflammatory, atherogenic environment is created by an increase in T-cell to macrophage ratio; release of inflammatory mediators like IL-1β, IL-6, TNF-α, and IFN-γ; and polarization to pro-inflammatory M1 macrophages with upregulation of C-X-C Motif Chemokine Ligand 9 (CXCL9). Lastly, vulnerability to plaque rupture is intensified by ICI-therapy dysregulating T-regs from mediating the fibrous cap formation. [Made using Biorender.com].

The stability of an atherosclerotic plaque determines its propensity to rupture resulting in a subsequent thrombus and cardiovascular complications like myocardial infarction or ischemic stroke. Regulatory T cells (Tregs) play an important role in maintaining these atherosclerotic lesions by secreting anti-inflammatory cytokines like IL-10 and TGF-β. This allows for the inhibition of Th1 cells and reduced macrophage activation, which allows for a tough fibrous cap to form (121). However, ICI-therapies dysregulate the function of T-regs, thus attenuating the anti-atherosclerotic protective effects (101). Magrolimab is an anti-CD47 monoclonal antibody that is a checkpoint inhibitor for macrophages. Although not in clinical practice, Magrolimab had a unique anti-atherogenic effect in human and animal trials likened to a potential induction of tumor cell phagocytosis (122). This study pointed towards the pro-atherogenic effects of CD47 in forming the necrotic core of apoptotic lipid-laden cells, the hallmark of atherosclerotic plaque formation (123). Figure 3 summerizes mechanistic pathways of immune checkpoint inhibitor (ICI)-induced endothelial dysfunction and accelerated atherosclerosis.

#### 3.2.2 Vasculitis

Vasculitides are a group of rare diseases characterized by inflammation of the blood vessels, causing them to narrow, weaken, or stretch. Central to the mechanism of vasculitis is immune-mediated damage to the blood vessels' endothelium (124). Immune checkpoint inhibitor (ICI)–associated vasculitis typically occurs in older adults, with a median age of 65 years (IQR 53–70), and shows a male predominance (60%). Melanoma was the most common underlying malignancy, accounting for 45% of reported cases. Most events were linked to anti–PD-1 therapies (58%), followed by combination ICI regimens (30%) and anti–CTLA-4 agents (11%). Vasculitis subtypes included large-vessel vasculitis, antineutrophil cytoplasmic antibody–associated vasculitis, and predominantly non-LVV/AAV forms, most commonly single-organ vasculitis (88%). Among LVV cases, giant cell arteritis was the leading presentation. Vasculitis generally developed a median of 12 weeks (IQR 4–32) after initiation of ICI therapy. Treatment responded well to initial glucocorticoid therapy and ICI discontinuation (125).

Evidence highlights a central role of PD-1 and CTLA-4 pathways in vasculitis pathophysiology, with genetic polymorphisms in these pathways associated with vascular T-cell hyperactivity. The efficacy of abatacept, a CTLA-4–IgG1 fusion protein, in treating temporal arteritis further supports this mechanism. Reduced PD-1 expression promotes inflammatory signaling and recruitment of CD4^+^ T cells, macrophages, and multinucleated giant cells in medium- to large-sized arteries, suggesting that immune checkpoint inhibitor therapy may recreate a vasculitis-prone immune environment (18).

Cellular infiltration patterns of small vessel vasculitis in biopsy specimens demonstrate massive infiltration of antigen-independent activated CD8^+^ T cells (positive for Granzyme B and TIA-1, negative for CD25) along with CD163^+^ macrophages, while CD4^+^CD25^+^ Treg cells are notably absent or reduced. These findings were taken from a patient with ICI-related tubulointerstitial nephritis (126). This provides more quantitative evidence for the dysregulation and pattern of vascular infiltration, which also mimics the pattern of myocarditis.

### 3.3 Arrhythmogenic mechanisms in ICI-associated cardiotoxicity

Cardiac arrhythmias have emerged as an important category of cardiovascular immune-related adverse events associated with immune checkpoint inhibitors (ICIs), reflecting the expanding recognition of cardiotoxicity beyond myocarditis in patients receiving immunotherapy. Analysis of pharmacovigilance data from the U.S. Food and Drug Administration Adverse Event Reporting System (FAERS) identified 81,643 adverse events related to ICIs and 111,384 reports involving cardiac arrhythmias between 1 January 2017 and 30 June 2021, highlighting the substantial clinical burden and growing relevance of rhythm disturbances in this population. Among reported preferred terms (PTs), complete atrioventricular block, atrial fibrillation, and sudden death were identified as overlapping and clinically significant arrhythmic outcomes. Sudden death was the most frequently reported event and represented one of the strongest safety signals (IC₀₂₅ = 1.90), underscoring the potential severity of ICI-associated electrophysiological complications (127).

PD-1 inhibitors (nivolumab and pembrolizumab), PD-L1 inhibitors (atezolizumab and avelumab), and combination therapies (ipilimumab plus nivolumab) were associated with significant cardiac arrhythmias. With CTLA-4 monotherapies being an interesting exception (127). Each of these demonstrated significant associations with complete atrioventricular block, cardiac arrest, tachycardia, and atrial fibrillation (128). Oftentimes, these arrhythmias are concurrent with other cardiotoxicities, including cardiac failure, coronary artery disease, myocardial disorders, pericardial disorders, and cardiac valve disorders. Temporal analysis further revealed that arrhythmias typically occur early after treatment initiation, with median times to onset ranging from 18 days with avelumab to 56 days with cemiplimab, while ipilimumab, nivolumab, pembrolizumab, atezolizumab, and durvalumab demonstrated median onset times between 25 and 47 days (127). These findings indicate that arrhythmic complications often develop within the first two months of therapy, emphasizing the importance of early cardiovascular monitoring and heightened clinical awareness during the initial phases of ICI treatment.

While the mechanisms of ICI-related arrhythmias are poorly understood, the association with other cardiovascular complications suggests that these arrhythmias may be secondary to structural changes caused by ICI-induced heart failure or myocarditis (129). ICI-induced cardiac fibrosis occurs through distinct mechanisms from acute myocarditis, involving both inflammatory and non-inflammatory pathways. Preclinical models demonstrate that short-term ICI therapy (10 days) induces myocardial fibrosis with increased expression of galectin-3, pro-collagen 1-α, and MMP-9, accompanied by upregulation of damage-associated molecular patterns (DAMPs), NLRP3 inflammasome, and MyD88 signaling. The NLRP3-MyD88 pathway appears central to cardiac fibrosis, with both anti-CTLA-4 and anti-PD-1 therapies increasing expression of fibronectin-EDA, S100/calgranulin, and galectin-3, along with systemic elevation of SDF-1, IL-1β, and IL-6 (107, 130). This cardiac fibrosis may result in underlying structural changes that predispose to cardiac arrhythmias. In particular, fibrotic tissue is vulnerable to reentry currents through the creation of tenuous interconnected myocytes. Additionally, fibrotic myocytes require significantly less myocyte activation to trigger a premature ventricular contraction, which can induce other ventricular arrhythmias (131, 132).

Besides these mechanisms, ICI-myocarditis may also result in damage to the atrioventricular (AV) and sino-atrial (SA) nodes, resulting in complete AV block or sick sinus syndrome. These are rare complications of myocarditis (133, 134). This can result from direct inflammation and damage of the cardiac conduction system from the SA node to the Bundle of His. Arrhythmias may also develop secondary to other cardiotoxic effects such as reduced left ventricular function (135).

Inflammation in general can promote cardiac arrhythmias through several indirect pro-arrhythmic mechanisms mediated by cytokines such as TNF, IL-1, and IL-6. All of which are significantly elevated during ICI-therapy (136). Although still an area of little research, there are several potential mechanisms that cytokine dysregulation or acute inflammation may predispose to arrhythmias. Firstly, acute inflammation and increasing circulating levels of cytokines may prolong the QTc interval, leading to the risk of other deadly arrhythmias like Torsades de Pointes (137). In addition, inflammation affects autonomic and thermoregulatory pathways by inducing fever and sympathetic nervous system activation through central and peripheral mechanisms, including hypothalamic inflammatory reflexes and stellate ganglion stimulation. These changes alter cardiac ion channel behavior, prolong action potentials, and promote intracellular calcium overload, collectively creating a substrate that increases susceptibility to cardiac arrhythmias (138).

Although experimental studies suggest that immune checkpoint inhibition may promote myocardial fibrosis through activation of DAMP-associated pathways, NLRP3 inflammasome signaling, and MyD88-dependent inflammatory cascades, evidence supporting fibrosis as a major driver of arrhythmias in ICI-treated patients remains limited. Current clinical observations primarily associate arrhythmias with active myocarditis, myocardial inflammation, and conduction system involvement rather than established fibrotic remodeling. Furthermore, most mechanistic data originate from animal models and circulating biomarker studies, while direct histopathological and longitudinal imaging evidence in humans is sparse. Consequently, whether myocardial fibrosis represents an independent arrhythmogenic substrate in ICI cardiotoxicity or a secondary consequence of inflammatory myocardial injury remains an important unresolved question requiring prospective investigation. Therapeutic strategies for managing ICI-associated cardiotoxicity are detailed in Table 2, which outlines the underlying molecular targets, classifies approaches as emerging or established, and indicates the preclinical or clinical trial phase.

**Table 2:** Current and emerging therapeutic strategies for immune checkpoint inhibitor–associated cardiovascular toxicity.

**Therapeutic Target/Strategy**	**Representative Therapies**	**Development Stage**	**Evidence Base**	**Potential Role in ICI Cardiotoxicity**
High-dose corticosteroids	Methylprednisolone, Prednisone	Current Standard-of-Care	Clinical guidelines, retrospective cohorts, multicenter studies (169, 229).	First-line treatment for suspected ICI myocarditis and other severe cardiovascular irAEs
Plasmapheresis	Plasmapheresis	Emerging Standard-of-Care	Clinical practice guidelines and case series (186, 230).	Potential first-line treatment for fulminant ICI myocarditis with immunosuppression risk
Second Line Therapies				
Broad immunosuppression for steroid-refractory disease	Mycophenolate mofetil	Emerging Standard-of-Care	Retrospective studies, case reports (16, 231, 232).	Escalation therapy in refractory myocarditis
	IVIG		Retrospective studies, case reports (16, 233).	
	Antithymocyte globulin		Retrospective studies, case reports (16, 234).	
	Tacrolimus		Retrospective studies, case reports (16).	
Immune-Reprogramming Therapies				
Immune Checkpoint Modulation	Abatacept	Clinical Investigation	Human case reports, observational studies, ongoing clinical trials (e.g., ATRIUM) (222)	CTLA-4 agonist (dampen excessive T cell activation)
JAK/STAT Pathway Modulation	Ruxolitinib, baricitinib	Clinical Investigation	Human case reports and early prospective studies (235, 236).	JAK inhibitors. (shift macrophage toward M2 phenotype)
Metabolic Pathway Inhibitor	NG52	Preclinical	Murine model with autoimmune myocarditis (187).	PGK1/PDHK1 Inhibition. (Shifts toward anti-inflammatory T cells)
	2-DG PFK158	Preclinical	Murine model with atherosclerosis (237).	Attenuate atherosclerosis via Glycolysis Inhibition (188, 189). (downregulate Th17, upregulate T-regs)
	Rapamycin	Preclinical	Murine model with autoimmune myocarditis (238).	mTOR Inhibition (85). (suppresses pro-inflammatory metabolism)
Pyroptosis	Z-DEVD-FMK	Preclinical	Murine model (239, 240).	Gasdermin Pathway/ Caspase-3 Inhibition (203). (downregulates GSDME activation which downregulates IL-1β, TNF-α)
Chemokine Axis	Anti-CXCR3	Preclinical	Murine model with ICI- myocarditis (204).	CXCL9/10 Macrophage and CXCR3 T-cell (204). (depletion of pro-inflammatory macrophages, NF-κB pathway reduction)
	CXCL10 Ligand Blockade			
	Clondronate Liposomes			
Signal Pathway Modulation	Low-intensity pulsed ultrasound (LIPUS)	Preclinical	Murine model with ICI- myocarditis (191).	HIPPO Pathway (85, 191). (Control Th17 vs. Treg balance)
	ONX 0914	Preclinical	Murine model with viral- myocarditis (196)	Immunoproteasome Inhibition (86). (downregulate Th1/Th17, upregulate T-regs)
	Tocilizumab, Crocin	Clinical Investigation	Human case reports (192, 241), murine model of ICI myocarditis (242).	IL-6 Inhibition (192). (prevent downstream inflammatory response)
	Fenofibrate	Preclinical	Murine model with ICI- myocarditis (243).	PPARα Activation (199). (downregulate Th17, upregulate T-regs)
	Y-27632 (ROCK inhibitors)	Preclinical	Murine model with ICI- myocarditis (244).	ROCK Pathway Inhibition (38684719, 32695015). (downregulate IL-1β and inflammation)

The table summarizes the therapeutic strategies for ICI-associated cardiotoxicity, detailing their developmental stages as emerging or established approaches, or by their preclinical and clinical trial phases, alongside supporting evidence and potential molecular targets.

## 4 Clinical surveillance and management of ICI-associated cardiotoxicity

Immune checkpoint inhibitors (ICIs) have transformed modern oncology by enhancing T-cell–mediated antitumor immunity and improving survival across multiple malignancies. However, immune activation also disrupts normal immune tolerance, resulting in immune-related adverse events (irAEs) that can affect nearly any organ system. Cardiovascular irAEs, although uncommon, are increasingly recognized as clinically significant due to their potential severity and high mortality, particularly in cases of myocarditis. Current guidance on surveillance and management remains largely consensus-based because prospective data are limited, but evolving recommendations from oncology and cardiology societies emphasize early recognition, structured monitoring, and rapid immunosuppressive treatment to reduce morbidity and mortality.

### 4.1 Clinical indications and cardiotoxicity associated with ICI combination therapies

Immune checkpoint inhibitors are commonly combined with other therapies to improve efficacy against other tumors. These include chemotherapy, other targeted therapies, and dual ICI therapies. With each of these different combination therapies, there are potential exacerbated cardiac effects. The following section will summarize the current indications for these dual therapies and the potential cardiotoxic effects that may be observed.

ICI and chemotherapy have synergistic and complementary anti-tumor mechanisms. The various mechanisms of chemotherapy interfere with the ability of cancer cells to grow, divide, and multiply, oftentimes leading to cancer cell apoptosis. Upon apoptosis, tumor cells release damage-associated molecular patterns (DAMPs) that can be recognized by immune cells to provide anti-tumor immunity (139). In particular, these combined strategies are becoming first-line indications in treating non-small cell lung cancers, which have a proven effectiveness over chemotherapy alone (140). Other notable indications for combined therapies include renal cell carcinoma, bladder cancer, triple-negative breast cancer, head and neck cancer, and gastric and esophageal cancers (141–144). However, both chemotherapies and ICI therapies have notable and well-documented cardiotoxicities even when given independently (12, 145, 146). That being said, combined therapies utilizing both chemotherapies and ICI-therapy have not displayed any significant increase in cardiovascular toxicity compared to chemotherapy alone (147, 148). Though ICI therapy with doxorubicin specifically has been demonstrated in a small patient population to have an increased risk of developing left ventricular dysfunction compared to doxorubicin alone (149).

Likewise, ICI and targeted therapies are also a powerful new therapeutic option for cancer, where the targeted therapies are able to directly modulate the tumor microenvironment. Vascular Endothelial Growth Factor (VEGF) pathway inhibitors or anti-angiogenics inhibit tumor cells' angiogenesis but can also modulate immune cell activity (150–152). Typically, VEGF inhibits T-cell trafficking across tumor epithelium, suppresses dendritic migration, and expands Tregs (153–156). Thus, the targeted anti-VEGF therapies can facilitate anti-tumor immune response and be synergistic with ICI therapy (152). This has been demonstrated to be effective in renal cell carcinoma and advanced hepatocellular carcinoma (157, 158). VEGF inhibitors are associated with significant cardiotoxicity, including hypertension and cardiac dysfunction (159). Moreover, combined ICI and anti-VEGF therapies are associated with a significantly higher risk for grade 3–4 hypertension, arterial thromboembolic events, and grade 3–4 cardiac disorders. Notably, patients with the combination therapies were 2.69 times more likely to experience a fatal cardiovascular event. Although these therapies provide potential avenues of therapy in very aggressive cancers, implementation of these therapies must be exercised with careful monitoring for vascular complications and blood pressure (160). Other notable synergistic targeted therapies are BRAF/MEK inhibitors in the MAPK pathway. BRAF/MEK inhibitors specifically target the pathways of uncontrolled cancer cell division but can upregulate HLA protein expression and downregulate immunosuppressive cell types (161). Thus, this can increase immune sensitivity. In particular, combined ICI and BRAF/MEK inhibitors are utilized in metastatic melanoma (162, 163). BRAF/MEK inhibitors have notable cardiotoxicity through left ventricular systolic dysfunction and QT prolongation (164). At this moment, the efficacy and safety of this combined therapeutic approach are still being studied through the IMspire150 trial (165). Thus, there is still little evidence on the cardiotoxic effects of a combined therapy.

Lastly, dual ICI utilizing both anti-CTLA-4 and PD-1/PD-L1 agents can provide a synergistic mechanism to increase anti-tumor immunity at different stages of the immune response. Dual ICI therapies are now utilized in treating melanoma, renal cell carcinoma, hepatocellular carcinoma, malignant pleural mesothelioma, and esophageal squamous cell carcinoma (166). Moreover, dual ICI-therapy has a significantly greater incidence of myocarditis at 1%–2% in dual ICI therapy compared to 0.75% with single-agent ICI therapy (167). Likewise, the rates of other cardiac irAEs are significantly greater in patients treated with Dual ICI, including atrial fibrillation, heart failure, and pericardial effusion (168).

### 4.2 Surveillance strategies

Existing guidelines recommend individualized surveillance for ICI-associated toxicities, guided by baseline cardiovascular risk and treatment regimen, as standardized monitoring protocols are not yet fully established. Routine screening with electrocardiography, cardiac biomarkers (such as troponin and natriuretic peptides is recommended for high risk patients including those on combination therapy and pre-existing cardiovascular disease (169, 170). When cardiovascular toxicity is suspected with worsening symptoms, prompt evaluation should include additional chest imaging, and echocardiography can be utilized to assess ventricular function (171–173). More definitive testing includes cardiac MRI and endomyocardial biopsy (174, 175). Patients with suspected cardiovascular irAEs should be hospitalized, monitored on telemetry, and evaluated collaboratively by cardiology or cardio-oncology specialists. Although routine screening schedules remain debated, serial ECG and troponin monitoring have been explored in surveillance studies as a strategy for early detection of subclinical disease (70, 176).

The European Society of Cardiology currently recommends troponin testing r for patients receiving ICI with Class Ia recommendations (177). The optimal Cardiac Troponin test between cardiac troponin I (CTnI) and cardiac troponin T (CTnT) remains a gap in research. Oftentimes, the choice of the test is determined on an institutional basis. CTnT has a lower specificity and higher false positive rate than CTnI due to the possibility of expression in patients with myositis (178). However, this may also provide better prognostic value given that ICI cardiotoxicity is oftentimes a systemic disease that affects the skeletal muscles too (179). Moreover, there has been growing concern that troponin testing alone may overdiagnose myocarditis, resulting in unnecessary discontinuation of ICI therapy (180). Although not in routine standard of care, creatine kinase (CK) levels have demonstrated greater efficacy as a biomarker due to origin in the myocardium (181–183).

Elevated NT-proBNP has emerged as an important prognostic biomarker for risk stratification in patients with severe immune checkpoint inhibitor (ICI)–associated myocarditis, helping identify individuals at higher risk of adverse outcomes. While immunotherapy rechallenge may be feasible in carefully selected patients, it should be approached cautiously, particularly in those with a history of moderate-grade myocarditis, due to the potential for recurrence or worsening toxicity. Overall, these findings support biomarker-guided therapeutic escalation and emphasize the importance of shared decision-making to balance ongoing oncologic benefit with cardiovascular safety in patients experiencing ICI-related cardiac complications (184). Preliminary studies are now also focusing on incorporating more comprehensive immune profiling for myocarditis and more importantly characterize the severity of the myocarditis. Key markers include IL-6, CXCL9, CXCL10, CXCL13, VEGF-A, and sCD25. Moreover, low levels of CCL4 and CXCL12 were associated with high-grade myocarditis at accuracies of 78.6% and 82.1%, respectively (144). These profiles can provide a more nuanced perspective and a more in-depth classification system for myocarditis.

### 4.3 Therapeutic strategies

#### 4.3.1 Current standard of care

Management of irAEs is severity-based, typically following a graded framework similar to National Comprehensive Cancer Network recommendations. ICIs should be held immediately when cardiovascular toxicity is suspected and permanently discontinued for moderate to severe events. High-dose corticosteroids remain first-line therapy, with prednisone (1–2 mg/kg/day) or intravenous methylprednisolone (1 g/day) initiated early and tapered gradually over 4–6 weeks once biomarkers and cardiac function normalize. Patients who fail to improve within 24 h may require additional immunosuppressive therapies, including intravenous immunoglobulin, antithymocyte globulin, mycophenolate mofetil, tacrolimus, infliximab, or abatacept in refractory cases. Multidisciplinary management and careful monitoring for infectious complications are strongly recommended, and resumption of ICI therapy may be considered only in selected patients with mild toxicity after careful risk–benefit assessment (185).

#### 4.3.2 Plasmapheresis

Plasmapheresis represents a potential new therapeutic approach that is synergistic with glucocorticoids. Although only implemented in several cases, the plasma exchange has potentially improved efficacy though larger trials are still required. The plasma exchange rapidly removes many of the antigens, antibodies, immune complexes, and cytokines that drive myocarditis while also mitigating other complications like “cytokine storm.” However, this approach is contraindicated in the setting of severe decline of cardiac function (186).

#### 4.3.3 Emerging immune-reprogramming therapies

Immune reprogramming is emerging as a new line of therapies for cardiac irAE focused on targeting T-cell and macrophage function and differentiation to provide precise modifications to immune cell behavior. The ultimate goal is to reduce harmful inflammation while preserving immune response and anti-tumor effects. As such, the target of immune reprogramming therapies fall into several key targets: metabolic reprogramming, signaling pathway modulation, immune checkpoint modulation, and pyroptosis regulation. It is important to note the novelty of these therapies and that these therapies are still actively undergoing research for efficacy and safety within animal models.

Pro-inflammatory Th17 and Th1 cells are dependent on glycolytic processes for energy. Thus, inhibition along the glycolytic process can attenuate T cells. Research has found that phosphoglycerate kinase 1 (PGK1), a key enzyme in glycolysis that is often overexpressed in neoplastic cells, can be inhibited by NG52, thereby reducing inflammatory T cell activity and mitigating cardiac inflammation in cases of ICI-induced myocarditis (187). In similar fashion, 2-deoxy-D-glucose (2-DG) inhibits hexokinase of the glycolysis pathway, ultimately reducing IL-17 and increasing levels of anti-inflammatory regulatory T (Treg) cells (188). Likewise, metabolic reprogramming is also a viable target for macrophages. Therapeutic inhibition of PFKFB3 has shown promise in reducing atherosclerosis by reducing the progression of macrophages and mononuclear cells, which in turn reduces the plaque burden (189). mTor inhibition via rapamycin has also been shown to block the HIF-1α expression in Th17 cells (190).

Importantly, novel therapies have also focused on inhibiting immune signaling pathways. The HIPPO pathway is a highly involved pathway involved in CD4+ T cell activation in ICI-related myocarditis, which activates TAZ to allow for Th17 differentiation. Low-intensity ultrasound (LIPUS) is typically used for treating ischemia-induced cardiac dysfunction and ventricular remodeling. However, it has also been shown to decrease the expression of TAZ, downregulating the HIPPO pathway, and regulating Treg and Th17 differentiation (191). The NF-kB drives the production of pro-inflammatory cytokines like IL-1β, IL-6, and TNF-α, which have downstream effects strongly associated with many of the cardiac irAEs (192).

Y-27632 is a Rho kinase (ROCK) inhibitor that targets ROCK2, a kinase that is involved in the differentiation of dendritic cells, macrophages, and natural killer cells. ROCK is further involved in several pathways like the JAK/STAT and RhoA/ROCK/HIF-1a signaling pathway (193, 194). Other JAK/STAT pathway inhibitors, including ruxolitinib and baricitinib, have another benefit in polarizing macrophages into the M2 phenotype, which downregulates inflammation and aids in tissue repair. Although not directly observed in ICI-induced myocarditis, this may be a potential target for future therapies. In the case of severe myocarditis, direct CTLA-4 agonists can also provide a direct reversal of the effect of ICI. The agonist binds to CD80 and CD86 on APCs and has downstream effects that downregulate the mTOR pathway (195).

Furthermore, ONX 0914, an immunoproteasome inhibitor, targets the Toll-like receptor (TLR) signaling pathway. Although not utilized in the context of ICI-induced myocarditis, immunoproteasome inhibitors have demonstrated effectiveness in other forms of acute viral myocarditis in mouse models (196). Immunoproteasomes promote Th17 and Th1 cells while impairing Tregs, which exacerbate cardiac inflammation and fibrosis (197). Immunoproteasome inhibitors increase PD-1 expression on CD4^+^ T cells, counteracting the activity of immune complex inhibitors, working to protect the heart from excessive inflammation (198). Similar to other potential therapies that target T cell function and differentiation, suppression of the immunoproteasome also modulates T cell activity, with cell polarization shifting from the pro-inflammatory Th1 and Th17 phenotypes to the cardio-protective Treg cells (196). This occurs via reduction of pro-inflammatory cytokines, ultimately resulting in less leukocyte migration into cardiac tissue (86).

Interestingly, fenofibrate, a drug classically used in cholesterol management, has been found to also have influence on T cell behavior. Its upregulation of peroxisome proliferator-activated receptor alpha (PPAR-ɑ) signaling pathway ultimately also promotes the functioning of Treg cells, which are protective against excessive inflammation, therefore reducing myocarditis (199). A caveat to these T-cell targeting therapies is the potential loss of anti-tumor activity. ICI therapy relies on T cell targeting of malignant cells; this immune response can ultimately be blunted by therapeutics treating ICI-induced myocarditis (200). Interestingly, fenofibrate has also demonstrated independent antitumor properties through several complex pathways that result in cell-cycle arrest and inhibition of tumor invasion (201).

Other potential forms of therapy include those aimed at reducing pro-inflammatory cytokine release, specifically ones related to the process of pyroptosis, or inflammatory lytic cell death. Caspase 3 is normally responsible for apoptosis, or cell death that is non-inflammatory. However, when in the presence of Gasdermin E (GSDME), caspase 3 cleaves GSDME, leading to cell lysis and release of pro-inflammatory cytokines (202). Experimental models suggest that caspase 3 inhibitor Z-DEVD-FMK reduces pyroptosis, therefore lowering levels of inflammation. This therapy can potentially diminish the immune response to ICI-induced myocarditis, preventing further inflammatory damage (203).

CXCL9 (C-X-C motif chemokine ligand)/CXCL10^+^CCR2^+^ (C-C motif chemokine receptor) macrophages and CXCR3hi (C-X-C motif chemokine receptor 3 high-expressing) CD8^+^ T cells are elevated in the heart of mice models with ICI-induced myocarditis (204). Targeting the interaction between CXCR3^+^ T cells and CXCL9/10-producing macrophages may have benefits in cases of ICI-induced myocarditis, reducing inflammatory cell infiltration into cardiac tissues (78). Targets in this approach include the use of anti-CXCR3 antibodies, blockade of the CXCL10 ligand, and use of clodronate liposomes to deplete pro-inflammatory macrophages (204, 205). Mechanistically, downregulation of CXCR3 led to decreased expression of CD49d, a transmembrane glycoprotein responsible for T-cell migration into damaged cardiac tissue. Similarly, signaling action of the NF-κB pathway is reduced, decreasing levels of pro-inflammatory cytokines and adhesion molecules responsible for immune cell attachment and transmigration (120, 204).

Despite substantial advances in the understanding of immune-cell reprogramming in ICI-associated myocarditis, the majority of proposed therapeutic strategies remain at the preclinical stage. Experimental studies have demonstrated promising effects of metabolic modulation, signaling pathway inhibition, macrophage reprogramming, immunoproteasome blockade, pyroptosis suppression, and chemokine-targeted therapies in reducing cardiac inflammation and promoting immune tolerance. The clinical application of these approaches is hindered by questions about preserving antitumor immunity, heterogeneous patient responses, and the scarcity of prospective biomarker trials. Consequently, while these approaches show mechanistic promise, they remain to be validated as established treatments for ICI-myocarditis. The therapeutic strategies for ICI-associated cardiotoxicity are summarized in Table 3, which highlights their developmental stages, preclinical and clinical phases, supporting evidence, and potential molecular targets The therapeutic strategies for ICI-associated cardiotoxicity are summarized in Table 3, which highlights their developmental stages, preclinical and clinical phases, supporting evidence, and potential molecular targets.

**Table 3:** Clinical characteristics, diagnostic evaluation, and management of major cardiovascular immune-related adverse events associated with immune checkpoint inhibitor therapy.

**Cardiovascular irAE**	**Incidence**	**Typical Clinical Presentation**	**Diagnostic Tools**	**Management Strategies**
Myocarditis	Approximately 0.5%–1.2%; higher with dual ICI therapy (0.75% single-agent vs. 1%–2% combination therapy) (16, 245, 246).	Fatigue, dyspnea, chest pain, palpitations, heart failure, arrhythmias, cardiogenic shock; typically occurs within 1–2 months of ICI initiation (169).	ECG, cardiac troponin I/T, NT-proBNP, echocardiography, cardiac MRI, endomyocardial biopsy (169)	Immediate ICI discontinuation, hospitalization, telemetry monitoring, high-dose corticosteroids (prednisone 1–2 mg/kg/day or methylprednisolone 1 g/day), escalation to second-line immunosuppression/immune-modulators for steroid-refractory disease (70, 169, 247).
Pericarditis/Myopericarditis	TriNetX study identified pericarditis (0.22%). Myopericarditis cases among ICI-treated patients with cardiac inflammatory syndrome is 0.89% (84, 248).	Chest pain, dyspnea, pericardial effusion, tamponade, concomitant myocarditis, fatigue (249).	ECG, inflammatory markers, echocardiography for effusion assessment, cardiac MRI, CT imaging when indicated (169, 249).	ICI interruption, corticosteroids, pericardiocentesis for hemodynamically significant effusions, multidisciplinary cardio-oncology management, rule out other etiologies of pericarditis (170, 249).
Arrhythmias	Atrial arrhythmias were most common (1.1% −8.2%) (250), ventricular arrhythmias and sudden cardiac death are second most common and especially associated with avelumab (128, 251). Complete AV block are rare often with myocarditis, high mortality (252).	Palpitations, syncope, dizziness, conduction abnormalities, sudden cardiac death (253).	ECG, ambulatory rhythm monitoring, telemetry, troponin testing, echocardiography, cardiac MRI to evaluate concomitant myocarditis (169, 173, 254).	Management of underlying myocarditis/inflammation, corticosteroids when immune-mediated, antiarrhythmic therapy, pacemaker implantation for AV block, standard heart rhythm management according to guideline-directed therapy (129, 254)
Vasculitis	Rare complication found mostly in case studies with a median time of onset after starting ICI was 4–7.2 months (255, 256)	Ischemic, neurologic and renal manifestation Constitutional symptoms, headache, visual symptoms, includes large-vessel vasculitis and ANCA-associated vasculitis (256, 257)	ESR/CRP, autoantibody testing (ANCA), vascular imaging (CTA, MRA, PET), tissue biopsy when feasible (125, 258).	ICI discontinuation, systemic corticosteroids, immunosuppressive therapy for severe cases, specialist-guided management depending on vasculitis subtype (125, 258, 259)
Atherosclerotic Complications (MI, Stroke, Coronary Events)	Approximately threefold increased risk of myocardial infarction, coronary revascularization, and ischemic stroke compared with matched controls with 5% incidence of ASCVD (113, 260)	Acute coronary syndrome, myocardial infarction, ischemic stroke, progressive ASCVD, thrombotic events (113, 261)	Lipid profile, ECG, cardiac biomarkers, coronary CT angiography, vascular imaging, conventional ASCVD evaluation (262, 263)	Aggressive cardiovascular risk-factor modification, statin therapy when indicated, antiplatelet therapy per standard guidelines, management of acute coronary syndromes and stroke according to established cardiovascular guidelines (113, 263, 264)

The table stratifies distinct cardiovascular toxicities by their typical clinical presentation, recommended diagnostic algorithms (including biomarkers, imaging, and endomyocardial biopsy criteria), and current evidence-based management strategies ranging from first-line corticosteroids to advanced immunomodulatory therapies.

### 4.4 Current knowledge gaps and future directions

Immune checkpoint inhibitor therapy has fundamentally reshaped the oncologic landscape, producing durable remissions across an expanding array of malignancies. Conversely, the broad activation of T-cell immunity, renders the cardiovascular system a collateral target. This review has synthesized current mechanistic and clinical evidence regarding ICI-associated cardiotoxicity, with particular attention to the underexplored roles of immune responses such as the complement system as potential amplifiers of immune-mediated cardiac injury. The central unanswered question is why fewer than1% of ICI-treated patients develop myocarditis. Significant obstacles and critical questions regarding the late onset and predictive biomarkers also remain largely unresolved.

#### 4.4.1 Susceptibility mechansims

Kalinoski et al., have demonstrated that cardiac myosin heavy chain–specific tissue-resident memory T cells (TRM cells) accumulate in pericardial tissue after cardiac injury and express high levels of PD-1. In a two-hit murine model, mice with prior ischemic injury were significantly more susceptible to anti-PD-1—induced myocarditis than naive mice (206). A parallel preclinical study in ESC Heart Failure found that hypertensive cardiac remodeling was essentially a prerequisite for ICI-induced lethal myocarditis, with virtually no inflammatory cardiac infiltration in mice without prior injury. Together, these results suggest a plausible mechanism for individual susceptibility: patients who carry a pre-existing cardiac immune memory, whether from prior MI, ischemic injury, or subclinical myocardial damage, may harbor a reservoir of autoreactive TRM cells that checkpoint blockade then unleashes. This also maps onto the clinical observation that patients with pre-existing cardiovascular disease show elevated myocarditis rates (207). Furthermore, Yu et al. identified a decrease in central memory CD4^+^ T cells (CD4^+^ TCM) in the peripheral blood of patients who developed myocarditis, with IL-15 emerging as a correlate and potential regulator. Restoring CD4^+^ TCM cells via IL-15 in an anti-PD-L1 tumor mouse model reduced CD8^+^ TEMRA expansion and attenuated cardiac fibrosis markers, suggesting that the balance between protective and pathogenic T-cell subsets, not simply the total T-cell activation level, determines individual susceptibility (208).

A retrospective multicenter cohort study (209) found genetic evidence that the HLA-A01:01–B08:01–C07:01 haplotype (the 8.1 ancestral haplotype) was significantly enriched in patients with early-onset immune-related myotoxicity, with an odds ratio of approximately 3.6 compared both to cancer controls and healthy donors. Genetic analysis of of a smaller, distinct Chinese population revealed an increased prevalence of HLA-DQB103:03, HLA-C01:02, and HLA-B52:01 in individuals who developed immune checkpoint inhibitor-induced myocarditis, myositis, and myasthenia gravis overlap syndrome (210). Both studies are modest in size and require multicenter validation, but HLA-typing now has a plausible evidence base as a future pre-treatment risk-stratification tool.

The knowledge gap this field most urgently needs to fill is a prospective, large-scale biomarker study that links baseline immune phenotyping (TRM cell abundance, HLA haplotypes, CD4^+^ TCM frequency, pre-existing cardiac TRM burden quantified by advanced imaging or liquid biopsy) to incident myocarditis across a broad ICI-treated cohort. An experimental translational study using hiPSC-derived cardiomyocytes from patients who did and did not develop myocarditis, currently underway at several centers, is an approach that may reveal cell-intrinsic differences in cardiomyocyte susceptibility to IFN-γ–mediated injury (211).

#### 4.4.2 Predictive biomarkers

Biomarker-based surveillance strategies for ICI-associated myositis also remain inadequately validated. Although cardiac troponin is the standard for monitoring, clinical practice varies between cTnI and cTnT. These isoforms carry distinct specificities and prognostic profiles, which are critically challenged in patients with concurrent myositis, emerging immune profiling panels incorporating cytokines such as IL-6, CXCL9, CXCL10, CXCL13, and sCD25 offer a more granular stratification of myocarditis severity. Crucially, integrating early complement activation fragments (e.g., plasma C3a and C5a) into these predictive panels could help identify patients at risk for hyperinflammatory tissue injury before irreversible myocardial damage occurs. However, both cytokine and complement-based biomarker strategies require prospective validation in large multicenter cohorts before entering clinical algorithms.

#### 4.4.3 Therapeutic strategies and challenges

Therapeutic strategies aside from high-dose corticosteroids remain largely anecdotal or mechanistically speculative. Immunomodulatory approaches—including glycolytic inhibition targeting pro-inflammatory Th17/Th1 cells, ROCK2 inhibition, JAK/STAT pathway modulation, CTLA-4 agonism, and caspase-3 inhibition to suppress pyroptosis—hold considerable conceptual promise but lack evaluation in prospective clinical trials for ICI-induced cardiotoxicity. A central therapeutic challenge is preserving antitumor immunity while mitigating cardiac inflammation; therapies that broadly suppress T-cell activation risk blunting the oncologic benefit that justifies ICI use in the first place. Precision approaches that selectively target pathogenic immune cell subpopulations infiltrating cardiac tissue, without global immunosuppression, represent the most clinically desirable avenue for future research. This clinical precision relies heavily on resolving the molecular mimicry hypothesis. While mechanistically elegant and supported by TCR sequencing data linking identical T-cell clones across tumor, skeletal muscle, and myocardium, mimicry has been complicated by findings where heart-expanded T-cell clones fail to recognize canonical cardiac autoantigens. The identity of putative cardiac autoantigen(s) other than α-myosin and troponin remains unresolved. Bridging this gap would transform molecular mimicry into a dual-purpose clinical tool: first, as a highly specific biomarker strategy utilizing peripheral blood TCR sequencing to intercept cross-reactive clones before clinical symptoms emerge; and second, as an antigen-specific tolerance induction strategy to selectively silence the cardiotoxic autoimmune response while entirely sparing systemic antitumor immunity.

Looking ahead, overcoming these challenges requires coordinated prospective registries that capture granular immunologic, imaging, and outcomes data across the full spectrum of cardiovascular irAEs. Mechanistic studies employing single-cell transcriptomics, spatial proteomics, and *ex vivo* complement assays in cardiac biopsy or peripheral blood specimens from affected patients will be necessary to move beyond correlational observations. Randomized trials of novel immunosuppressive agents, with cardiovascular endpoints and embedded biomarker substudies, are urgently needed. Finally, the growing use of next-generation checkpoint targets—including LAG-3, TIM-3, and TIGIT inhibitors, alone or in combination—will almost certainly expand the landscape of cardiovascular irAEs in ways that are not yet fully characterized. Ultimately, the safe expansion of ICI therapy to an ever-broader oncologic population depends on a mechanistic understanding deep enough to anticipate, detect, and treat its cardiovascular consequences with the same rigor applied to its antitumor effects. The complement system, B-cell humoral pathways, and precision immune reprogramming strategies represent three underexplored frontiers where meaningful progress may be achievable in the near term.
